# A Two-Year Ecological Study of Norway Rats (*Rattus norvegicus*) in a Brazilian Urban Slum

**DOI:** 10.1371/journal.pone.0152511

**Published:** 2016-03-25

**Authors:** Jesús A. Panti-May, Ticiana S. A. Carvalho-Pereira, Soledad Serrano, Gabriel G. Pedra, Josh Taylor, Arsinoê C. Pertile, Amanda Minter, Vladimir Airam, Mayara Carvalho, Nivison N. Júnior, Gorete Rodrigues, Mitermayer G. Reis, Albert I. Ko, James E. Childs, Mike Begon, Federico Costa

**Affiliations:** 1 Centro de Pesquisas Gonçalo Moniz, Fundação Oswaldo Cruz, Ministério da Saúde, Salvador, Brazil; 2 Institute of Integrative Biology, University of Liverpool, Liverpool, United Kingdom; 3 Centro de Controle de Zoonoses, Secretaria Municipal de Saúde, Ministério da Saúde, Salvador, Brazil; 4 Department of Epidemiology of Microbial Diseases, Yale School of Public Health, New Haven, Connecticut, United States of America; 5 Instituto de Saúde Coletiva, Universidade Federal da Bahia, Salvador, Brazil; Université Pierre et Marie Curie, FRANCE

## Abstract

The Norway or brown rat (*Rattus norvegicus*) is among the most ubiquitous of rodents. However, the lack of studies describing Norway rat populations from tropical areas have limited our understanding regarding their demography and seasonal dynamics. In this study, we describe seasonal pattern in the abundance, reproductive parameters, and morphometrics of Norway rat populations in Salvador, Brazil. Rodents were trapped over four seasonal trapping periods (2013–2014) from three valleys. A total of 802 Norway rats were trapped over the course of the study over 7653 trap-nights. Norway rat abundance was high, but there was no significant differences between seasons. The reproductive parameters (e.g. frequency of pregnant and lactating females) did not show statistical differences between seasons. Female rats collected in the rainy season were heavier and older than females from the dry season. Salvador rats had a high incidence of pregnancy and birth rate (estimated birth rate of 79 young per year) compared to previous studies. The information generated is critical for the understanding of the ecology of Norway rat, the main reservoir of *Leptospira* in Salvador. However, future studies examining the effect of rodent control programs aimed at reducing populations, and determining rates of recovery, will further clarify our understanding of population dynamics.

## Introduction

The Norway or brown rat (*Rattus norvegicus*) is among the most ubiquitous of rodents. It lives in close proximity to humans in cities and is the cause of extensive economic damage to farms, food products, industries, and households [1]. In relation to public health, this species is a reservoir for important zoonotic pathogens such as bacteria (e.g. *Leptospira interrogans*), viruses (e.g. Seoul virus), and helminths (e.g. *Capillaria hepatica*) [2–4].

Norway rats have a high reproductive rate and behavioral plasticity allowing infestations of rural and urban environments [5]. In urban areas, rats abound in habitats where accumulated human garbage and favorable burrowing sites are plentiful [5,6]. In temperate and tropical cities residential areas, most notably of low socioeconomic status, refuse and improperly stored food, lack of sanitation, abandoned properties and poor household conditions all contribute to abundant rodent populations [7–9].

Although the Norway rat has a worldwide distribution, most studies on rat populations have been focused in temperate areas [10–15]. These studies have shown that reproductive parameters vary significantly among seasons or habitats (e.g. rural vs urban). The lack of studies describing Norway rat populations from tropical areas, however, has limited our understanding regarding the comparative demography and seasonal dynamics of Norway rat. Older studies have suggested that rat populations vary in their reproductive status between seasons in tropical areas [16,17], but these studies were conducted without systematic methodologies. Seasonal differences are influenced by both biotic and abiotic factors of the environment (e.g. photoperiod, food supply, and weather) that interact to regulate their growth rate and reproductive development [15]. Additionally, morphometric differences between temperate and tropical rat populations, consistent with Bergmann’s rule, add a further complication to direct generalizations of rats sampled from different latitudes [18].

Knowledge of rodent ecology in urban environments is vital, in particular, for the understanding of rodent-borne disease transmission [10], in addition to informing control programs by helping define eradication units [19]. As tropical countries, notably Brazil, suffer from an inordinate burden of rodent-borne disease in urban environments, such as leptospirosis [20], the lack of detailed long term studies is of special significance. For example, studies in Salvador, Brazil, have identified that sighting of rats and indicators of rat activity in peridomestic areas are risk factors for acquiring leptospirosis [21,22]. Herein, we describe seasonal patterns in the abundance, reproductive parameters, and morphometrics of Norway rat populations in Salvador, Brazil.

## Materials and Methods

### Study Site

This study was conducted in Pau da Lima, a slum community (*favela*) situated in the periphery of the city of Salvador (692,819 km^2^ in area) in Northeast Brazil (12°55ˈ28.03ˈˈS, 38°28ˈ34.9ˈˈW). This community is a three-valley area of 0.17 km^2^, with ~ 3,717 inhabitants. This area is characterized by the absence of basic sanitization, free flowing sewers and poor housing conditions [23]. The area was selected based on the high annual incidence of asymptomatic *Leptospira* infection (37.8 per 1,000 person-years) and of severe leptospirosis cases, characterized by renal insufficiency and pulmonary hemorrhage (19.8 per 100,000 pop.) [23]. Additionally, a cohort study of human leptospirosis [23], and a case-control study [21], revealed a high proportion of households infested with Norway rats and that rat infestation was significantly associated with the risk of *Leptospira* infection.

The initial locations of the 150 sampling points were selected at random from spatial maps of the area. However, because of safety concerns due to drug violence and seasonal flooding, 42 points were inaccessible, so the final number of points was reduced to 108 (72%; S1 Fig). Valley 1 included 26 sampling points, valley 2 40 sampling points, and valley 3 42 sampling points. Each sampling point included a buffer area of 30 m^2^. The study team surveyed all peridomestic areas or vacant lots looking for signs of rodent infestation [21] and selected three trapping sites within each sampling point buffer area. Permission to trap around households was obtained from residents.

### Trapping Methodology

Rodents were trapped over four trapping campaigns (TC) from the three valleys as follow: TC1, May to August 2013 (winter rainy season); TC2, October to December 2013 (dry season); TC3, March to July 2014 (winter rainy season); TC4, October to December 2014 (dry season). During each trapping campaign, trapping was performed over three (TC1) or two (TC2–4) sessions, at least one month apart, at each of the 108 sampling points. To avoid oversampling, a different and randomly selected trapping site from the three within each buffer area was chosen every month. Two live traps (45 x 16 x 16 cm, Tomahawk) were set at these trapping sites for four (TS1 and TS4) or six (TS2 and TS3) consecutive nights.

Following a previously described methodology [3,18], traps were baited with slices of sausage and placed, in the case of house yards, alongside a wall or fence close to signs of rodent activity (fecal droppings, burrows or active runs), potential resources of food (exposed garbage or pet food) and harborage (poorly constructed dwellings trash piles, or harborage such as discarded vegetation or accumulations of wood etc.). In open spaces or vacant lots, traps were placed close to garbage accumulations, open sewers or in vegetative coverage. In a few open spaces where live trapping was terminated due to vandalism, two snap traps were used. Traps containing animals were placed in plastic bags for transport, and fresh apple slices were provided to avoid the dehydration of rats. All animal procedures and methods were carried out following biosafety protocols as previously described [24].

### Ethics statement

The ethics committee for the use of animals from the Oswaldo Cruz Foundation, Salvador, Brazil approved the protocols used in this study (protocol number 003/2012), which adhered to the guidelines of the American Society of Mammalogists for the use of wild mammals in research [25] and the guidelines of the American Veterinary Medical Association for the euthanasia of animals [26]. These protocols were also approved by the Yale University's Institutional Animal Care and Use Committee (IACUC), New Haven, Connecticut (protocol number 2012–11498).

### Data collection

Daily inspection of traps occurred each morning, and each trap was reported as open, closed with an animal trapped, closed but no animal trapped, bait removed or stolen, or damaged. When baits were removed they were resupplied each morning. The number of non-target species caught was corrected for, along with sprung but empty traps, when computing capture success rates. Thus, trap success was used to estimate the relative rat abundance as follows: number of rats trapped x 100/(number of effective traps x number of nights), where the number of effective traps each night was given by the number of fully functional traps minus half the number of traps that were sprung but empty or contained non-target species [27]. Stolen and damaged traps were excluded from this calculation.

After euthanasia, the species, sex, mass, and body length were recorded. In females, sexual activity was characterized by the presence of placental scars, pregnancy (number of embryos was recorded), or evidence of lactation. Sexual maturity in males was determined by convoluted seminal vesicles [28]. Also, the presence or absence wounds to the skin was recorded.

The von Bertalanffy equation is often used with reference to growth curves to convert weight to age for mammalian and in particular rodent populations [29]. We converted the recorded weights of the animals to ages using the von Bertalanffy equation. The parameters for the von Bertalanffy were found by fitting data deduced from the growth curve for male rats presented by Calhoun [30]. Also, we estimated the body condition using a ‘Scaled mass index’ (Smi) based on mass and body length, whilst accounting for the effect of age [31].

### Data analysis

The non-parametric Mann-Whitney-Wilcoxon test was used to compare the trap success and the number of embryos per pregnant female between seasons [32]. Additionally, the t test was used to compare mass, age, and Smi of rats between sexes. To determine whether the sex ratio varied from 1: 1 between seasons, a Chi-squared test with Yates’s correction was used [32]. Additionally, the mass and age of each rat was classified (mass in one of six 100 g mass classes and age in one of five 60 days classes) to determine whether the cumulative proportion of each class differed between seasons using the two sample Kolmogorov-Smirnoff test. These mass/age class divisions made these data comparable to reports from Baltimore where similar methods were used to define the demographics of a temperate-zone Norway rat population [12]. We compared the proportion of sexually active, pregnant and lactating rats between seasons using a Chi-squared test of homogeneity [32].

We used generalized linear models (GLM) to examine the relationship between age and Smi of rats (log transformed to satisfy the model assumptions) and season, sexual activity, and wound presence. A logistic GLM was used to examine the relationship between wound presence and season, age, Smi, sex, and sexual activity. Variables associated in univariate models with a P < 0.1 were included in multivariate models, and a backward elimination strategy and the Akaike’s Information Criteria (AIC) [33] were used to select the best adjusted model from amongst candidate models. The final model was that with the lowest value of AIC, but the simplest model with a ΔAIC < 2 compared to this model (indistinguishable explanatory power) was then selected based on the parsimony principle. For all models, specimens with missing values for any of the variables under evaluation were excluded. The Spearman’s rank correlation coefficient was used to identify correlated variables [34]. In all statistical analyses, the level of significance was P < 0.05 and the software R (R Development Core Team, Vienna, Austria) was used.

To estimate the incidence of births per adult Norway rat over the total sampling days, we used the formula [35]: *F* = *I* x *t/* 18, where *F* = frequency of pregnancy, I = incidence of visible embryos, *t* = sampling days, and 18 = the number of days embryos are visible out of the estimated 23.5 to 25.5 days of gestation [36]. Frequency of pregnancy was corrected with an estimated 3% of intrauterine loss of entire litters [35]. Using the solution for *F*, the total number of rats entering the population was estimated by multiplying by the average number of embryos per pregnant female, considering an average loss of embryos of 20.5% [37], and estimates of total reproductive values per year were then extrapolated by assuming values do not change over the sampling periods.

## Results

### Trapping Results and Characteristics of Norway Rats

A total of 893 mammals were trapped over the course of the study during the 7653 trap-nights (6935 Tomahawk and 718 snap trap-nights). Norway rats (*Rattus norvegicus*) were the dominant species (802 individuals, 89.8%), followed by opossums (*Didelphis aurita*) (82 individuals, 9.2%) while black rats (*R*. *rattus*) were rarely captured (9 individuals, 1%). Only opossums trapped during the study were released at the point of capture. Norway rats were trapped in 91.7% (99/108) of sampling points and trap success overall was 13.1%. Black rats and opossums were excluded from analyses.

Four hundred Norway rats were males and 393 females, not significantly different from parity (data from 9 individuals were excluded for analyses). The mean mass of males, 300.4 ± 5.7 g (± standard error) was significantly greater than the 267.0 ± 5.6 g of females (t = -4.18, P < 0.01).

Rats in age class 61–120 days were most abundant (63% of males and 53.2% of females). Male rats had significantly greater ages than females overall (t = -4.40, P < 0.01). The mean male age was 88 ± 2 days and that for females 77 ± 2 days. There was no significant difference in the mean Smi between sexes (t = -0.19, P = 0.84; males 260 ± 3 and females 259 ± 3). The percentage of males with skin wounds, 60.1% (231/384) was significantly higher than the 47.8% (181/379) in females (χ^2^ = 11.31, P < 0.01).

Among males, 82.9% (326/393) were sexually active, whereas in females at least one characteristic of sexual activity was observed in only 57.7% of animals (224/388). Of sexually active females, 50.5% (106/210) were pregnant, 86.9% (147/169) had placental scars, and 35.8% (76/212) were lactating (30.3% of lactating females were also pregnant). Among pregnant females, the median number of embryos was 10 (first quartile 8.5; third quartile 12).

### Reproductive Rate of Rats

The parameters for estimating the reproductive rate of Salvador rats were *I* = 0.505 (proportion pregnant), *t* = 132 sampling days and 18 was assumed to be the number of days embryos were detectable. The frequency of births was 3.7 over the sampling period (females produced an offspring every 3.7 days, on average), and using the median number of embryos per pregnant female (10), females produced a litter every 37 days, on average. When corrected for the estimated loss of 3% intra-uterine loss of entire litters (0.11) and 20.5% of embryos mortality (2.05) the average sexually mature female Norway rat gave birth to a viable offspring every 4.6 days, or 79 viable offspring when extrapolated to a year (pregnancy proportion and median number of embryos did not vary across seasons).

### Seasonality

As trapping campaigns 1 and 3, and 2 and 4 had similar population characteristics, data were pooled into rainy and dry seasons, respectively (see S1 and S2 Tables). The trap success of Norway rats did not differ significantly between seasons (Rainy = 13%, Dry = 11.9%, U = 68.5, P = 0.31). The sex ratio was not different from 1:1 in either season (Rainy: χ^2^ = 0.11, P = 0.74; Dry: χ^2^ = 0.00, P = 1.00; Table 1).

**Table 1:** Summary of population characteristics of Norway rats for comparison between seasons in Salvador, Brazil.

Characteristic	Season			
	n	Rainy	n	Dry
No. of rats (percentage)		459 (57.9)		334 (42.1)
Male		233 (50.8)		167 (50)
Female		226 (49.2)		167 (50)
Mean (SE) mass (g):				
Males	233	306.8 (7.5)	167	291.4 (8.6)
Females	226	282.9 (7.4)*	167	245.5 (8.4)
Mean (SE) age (days)				
Males	233	86 (3)	167	83 (3)
Females	226	81 (3)*	167	70 (3)
Mean (SE) Scaled mass index				
Males	233	245 (4)	167	280 (5)*
Females	225	248 (4)	167	274 (6)*
No. of sexually active males (percentage)	228	191 (83.8)	166	135 (81.3)
No. pregnant rats (percentage)†	119	60 (50.4)	91	46 (50.5)
Median (1Q–3Q) of embryos	57	10 (9–12)	46	10 (8–12)
Mean (SE) of embryos	57	10.3 (0.3)	46	9.9 (0.5)
No. of lactating (percentage) rats†	120	41 (34.2)	92	35 (38)
Pregnant		11 (26.8)		12 (34.3)
Trap success (1Q–3Q)	15	13 (10.1–15.7)	12	11.9 (10.9–13)

SE, standard error; 1Q, first quartile; 3Q, third quartile;

^†^, considering only sexually active females;

*, P < 0.05.

**Fig 1:**
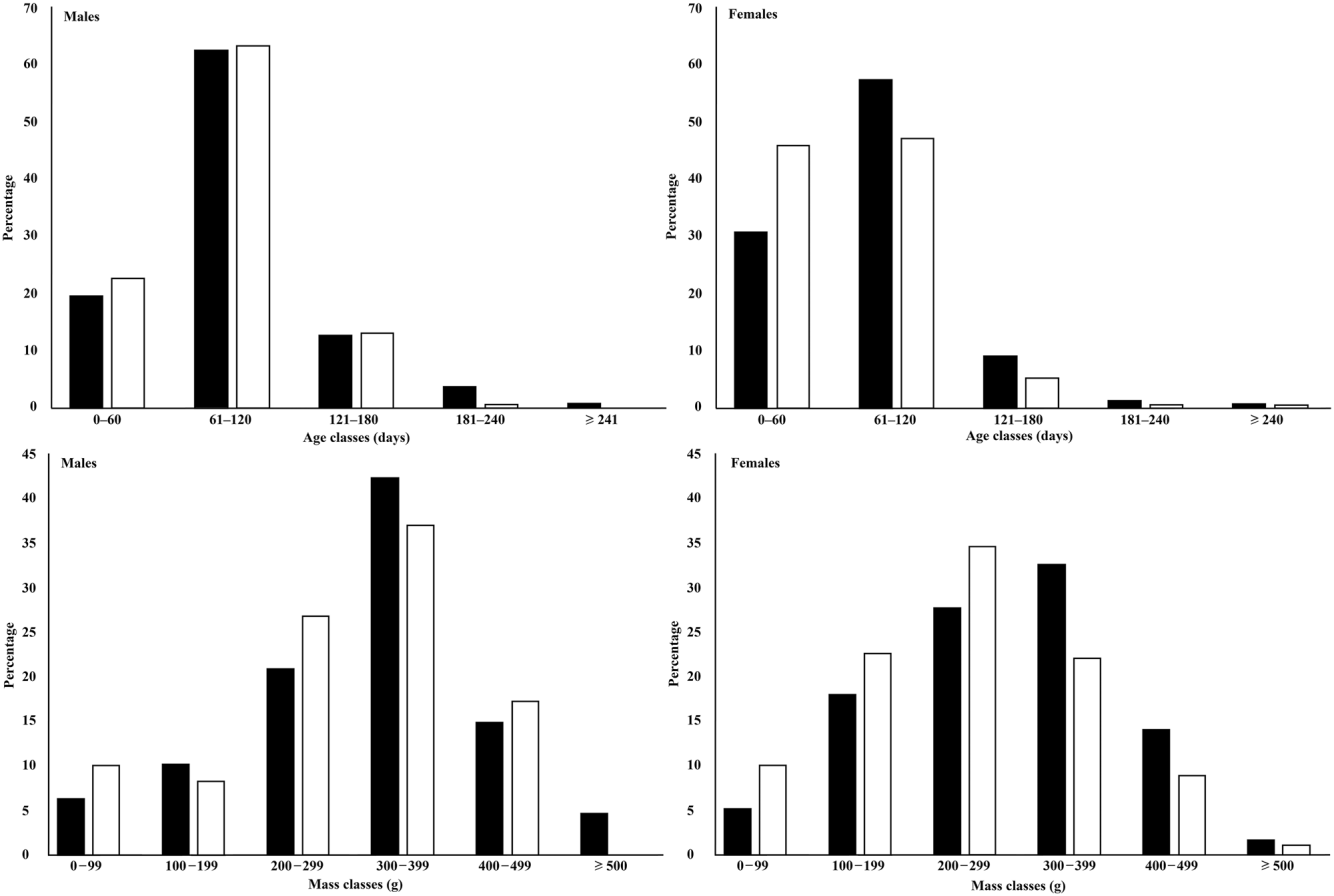
Seasonal comparison of age (*above*) and mass (*below*) classes for male and female Norway rats from Salvador, Brazil. Black bars for rainy season and white bars for dry season. Statistical differences were found in age and mass structure of females between seasons (P < 0.05).

Female rats collected in the rainy season were on average older than females from the dry season (t = -3.16, P < 0.01), but male rats showed no significant difference in age between seasons (t = -1.86, P = 0.06). Males and females had higher Smi values in the dry season compared with the rainy season (males, P < 0.01; females, P < 0.01; Table 1). There was a significant difference between the distributions of female age/mass classes between seasons (mass, D = 0.16, P = 0.01; age, D = 0.15, P = 0.02; Fig 1). In the dry season, females in age class 61‒120 days (46.1%) were more abundant than in the rainy season (31%; Fig 1). However, there was no significant difference between the male age/mass classes between seasons (Fig 1).

### Age

As age was significantly different between the sexes, the ages of males and females were modeled separately. On simple GLM, increased log age of males was associated with higher body condition (Smi), sexual activity and wound scores, and the final model, too, included Smi (β = 0.0002, 95% CI = 0.0001–0.0004), sexual activity (β = 0.30, 95% CI = 0.26–0.33), and wounds (β = 0.11, 95% CI = 0.08–0.14). Log female age was associated with rainy, an increase of Smi, sexual activity and wounds, and the final model also included the season (β = 0.06, 95% CI = 0.03–0.09), Smi (β = 0.0003, 95% CI = 0.0001–0.0005), sexual activity (β = 0.17, 95% CI = 0.14–0.20), and wounds (β = 0.08, 95% CI = 0.05–0.11) (See S3 Table).

### Body Condition Based on Scaled Mass Index

On simple GLM, increased log Smi was increased in the dry season, and with age, sexual activity and wounds, but the final model only included age (β = 0.0004, 95% CI = 0.0001–0.0006) and season (β = 0.02, 95% CI = 0.01–0.04) (see S3 Table).

### Skin Wounds

With simple logistic GLM, wound presence in males was associated with age and sexual activity, and the final model also included both variables: age (OR = 1.03, 95% CI = 1.02–1.04) and sexual activity (OR = 2.35, 95% CI = 1.06–5.22). In females, wound presence was associated with age and sexual activity and in particular was greater in lactating females but not pregnant females. The final model included age (OR = 1.02, 95% CI = 1.01–1.03) and sexual activity (OR = 2.06, 95% CI = 1.26–3.36) (See S3 Table).

## Discussion

Tropical Norway rat populations are a nuisance, a threat to infrastructure and a public health risk due to the pathogens they maintain [2–4]; however, ecological studies focusing on urban Norway rat populations are largely restricted to temperate areas [10,12,14,15,38]. To our knowledge, no previous study has described in detail the seasonal patterns in population characteristics (e.g. demography, morphometric and reproductive status and reproductive rate) of Norway rats in a tropical urban location.

Salvador male rats were significantly older on average than females. Using mass as a proxy of age to compare with previous works, the figures were 291–307 g according to season in males, and 246–283 g according to season in females (Table 1). A previous study limited to the rainy season in Salvador, using data pooled from three rodent populations, did not find differences in the mass between sexes [18]. In temperate Baltimore, male rats were significantly heavier (325 g) than females (294 g) [12,39]. These differences may be influenced by weather patterns, phenotypical response to different environmental [39] and the natural attribute of sexual dimorphism; male Norway rats grew more quickly than females in both rural and city populations of rats from Baltimore [12,35,40,41]. In addition, we found that body condition (Smi), sexual activity and wounds also increased with age, which is consistent with results from an urban population in Canada [10].

Age and mass among female rats varied between seasons, with a decrease in age and mass during the dry season. It has been suggested that seasonal differences in mass structure, frequently used as proxy of age [3,40,42], are the result of different growth rates [12,39,43], differential mortalities [41] and migrations [11]. During the rainy season, abundant fruits and small invertebrates augment food resources [44], in addition to foods supplied by human garbage and pet food [21]. The increase in vegetation volume during the rainy season also provides additional cover and could thus reduce predation by dogs or cats (although undocumented), reducing mortality rates.

Skin wounds were more common in older animals, males, and sexually active rats, which is similar to results reported in North America [10,12]. As a species that lives in groups, dominance is a determinant of reproductive success in Norway rat populations [45], and aggression among males when competing for estrous females can result in wounding. In contrast, wounding among females occurs at lower frequencies and intensities than in males, being associated with maternal aggression mainly during the first days of lactation [46,47] which is consistent with the high proportion of lactating females with wounds in our study. Previous studies have reported that prevalence and loads of *L*. *interrogans* and presence of Seoul virus are associated with skin wounds in Norway rats [3,48]. Intra-specific aggression particularly in older males and lactating females could play a key role on *Leptospira* transmission.

The estimated frequency of births among Salvador adult female rats (3.6) was greater than that the 3.3 reported from wild urban rats in Baltimore [35]. Furthermore, the estimated interval between litters in Salvador (37) was far smaller than the 60–65 days reported in USA [35,49]. The high proportion of pregnant female rats and the short interval between pregnancies resulted in an estimated birth rate of 79 young per year compared to the estimate of 50.3 from Baltimore; an increase of 57.1%. The reasons for this marked difference are unknown but several factors such as a stable climate, abundant food resources or polyandry are likely to contribute to the greater fecundity of tropical rats in Salvador [21,50].

The reproductive rate and size of a rodent population is dependent of factors such as sufficient food, water, and harborage [51]. In tropical regions, seasonal reproduction of small mammals is related to rainfall and food availability [44]. In Salvador, the same proportion of females were pregnant in both seasons, indicating the absence of modifying effects of annual cycles of rainfall (see below). The environment of the study area was highly stable during the trapping period, with regard to temperature (average temperature: rainy season, 24.6 ± 1.6°C; dry season, 25.5 ± 1.0°C) but rainfall varied markedly (cumulative rainfall: rainy season, 1661.1 mm; dry season, 856.9 mm). The frequency of pregnancy varies greatly over the year in tropical and temperate locations. For instance, significant variations in the frequency of pregnancy, with peaks after the rainy season, have been described in Norway rats from tropical regions of Africa [16,17]. In temperate urban Baltimore, USA, high levels of pregnancy have been reported from February to June (~ 30–50% pregnant for adult rats > 200 g), but prevalence showed a decline in July (~ 28–38%) [14]. Another study in Baltimore, reported that the percentage of pregnant adult females varied greatly throughout the year with a peak in April (~ 80%) and minimal percentages in January and November (~ 15%) [12]. However, the mean number of embryos per female rat in Baltimore (10.1–10.5) [12,14] was similar to our median value of 10.1. In addition, urban *R*. *norvegicus* had a significantly higher number of embryos (10.1) than those of farm or rural rats (8.2) [14], suggesting that the availability of human generated high quality resources in different environments influence fecundity.

Similar proportions of sexually active males were found in the rainy season (83.8%) and the dry season (81.3%). Studies in temperate regions have shown that reproductive activity of male Norway rats occurs throughout the year without significant changes between seasons [12,52]. A detailed study in Baltimore rats reported no seasonal changes in the size of testes, seminal vesicles, ventral prostate, Cowper’s gland, and presence of sperm [52]. These studies suggest that temperature and rainfall do not influence the male Norway rat fertility.

We recognize several limitations to this study. Only two or three months were sampled per season and sampling was restricted to a single slum community. In many of the existing studies, rodent sampling has been carried out every month for at least one year [12,14,16,40]. In temperate areas, some studies have reported that reproductive patterns in rat populations vary between habitats, with rats captured from urban sites showing higher reproductive characteristics than their rural or semirural counterparts [12,14]. Variation in size and reproductive rates among Norway rats inhabiting rural locations in tropical climates deserves additional attention. Previous studies of tropical Norway rats from Africa suggest that reproductive variation occurs throughout year [16,17], but differences in environments sampled and trapping methods make direct comparisons difficult. As the existing data from tropical regions are so scarce it would be advisable to conduct further studies in order to improve our understanding of the dynamics of urban *R*. *norvegicus* populations from other locations.

These results indicate that urban Norway rat populations from Pau da Lima reach high abundances and maintain similar reproductive rates during the dry and rainy seasons. The information generated is critical for the understanding of the ecology of Norway rat, the main reservoir of *Leptospira* in Salvador, the construction of mathematical models predicting environmental leptospiral contamination and consequently the risk of leptospirosis transmission [48]. Future studies examining the effect of other factors, such as rodent control programs aimed at reducing populations and determining rates of recovery will clarify our understanding of population dynamics but also provide information about pathogen transmission.

## Supporting Information

S1 Fig Spatial distribution of sampling points (dark dots) for Norway rats in Pau da Lima, Salvador, Brazil. (TIF)

S1 Table Summary of population characteristics of Norway rats for comparison between trapping campaigns (TC) 1 and 3. (DOCX)

S2 Table Summary of population characteristics of Norway rats for comparison between trapping campaigns (TC) 2 and 4. (DOCX)

S3 Table AIC values for the four best models for age, scaled mass index (Smi), and wounds. The final models were selected by principle of parsimony.
(DOCX)

## References

[1] Pimentel D, Zuniga R, Morrison D. Update on the environmental and economic costs associated with alien-invasive species in the United States. Ecol Econ. 2005;52: 273–288.

[2] Easterbrook JD, Kaplan JB, Vanasco NB, Reeves WK, Purcell RH, Kosoy MY, et al A survey of zoonotic pathogens carried by Norway rats in Baltimore, Maryland, USA. Epidemiol Infect. 2007;135: 1192–1199. 17224086 10.1017/S0950268806007746 PMC2870671

[3] Costa F, Porter FH, Rodrigues G, Farias H, de Faria MT, Wunder EA, et al Infections by *Leptospira interrogans*, Seoul virus, and *Bartonella* spp. among Norway rats (*Rattus norvegicus*) from the urban slum environment in Brazil. Vector Borne Zoonotic Dis. 2014;14: 33–40. 10.1089/vbz.2013.1378 24359425 PMC3880909

[4] Himsworth CG, Bai Y, Kosoy MY, Wood H, DiBernardo A, Lindsay R, et al An investigation of *Bartonella* spp., *Rickettsia typhi*, and Seoul Hantavirus in rats (*Rattus* spp.) from an inner-city neighborhood of Vancouver, Canada: Is pathogen presence a reflection of global and local rat population structure? Vector-Borne Zoonotic Dis. 2015;15: 21–26. 10.1089/vbz.2014.1657 25629777

[5] Vadell MV, Gómez Villafañe IE, Cavia R. Are life-history strategies of Norway rats (*Rattus norvegicus*) and house mice (*Mus musculus*) dependent on environmental characteristics? Wildl Res. 2014;41: 172–184.

[6] Orgain H, Schein MW. A preliminary analysis of the physical environment of the Norway rat. Ecology. 1953;34: 467–473.

[7] de Masi E, Pino FA, Santos M das GS, Genehr L, Albuquerque JOM, Bancher AM, et al Socioeconomic and environmental risk factors for urban rodent infestation in Sao Paulo, Brazil. J Pest Sci. 2010;83: 231–241.

[8] Langton SD, Cowan DP, Meyer AN. The occurrence of commensal rodents in dwellings as revealed by the 1996 English House Condition Survey. J Appl Ecol. 2001;38: 699–709.

[9] Childs JE, McLafferty SL, Sadek R, Miller GL, Khan AS, DuPree ER, et al Epidemiology of rodent bites and prediction of rat infestation in New York City. Am J Epidemiol. 1998;148: 78–87. 9663407 10.1093/oxfordjournals.aje.a009563

[10] Himsworth CG, Jardine CM, Parsons KL, Feng AYT, Patrick DM. The characteristics of wild rat (*Rattus* spp.) populations from an inner-city neighborhood with a focus on factors critical to the understanding of rat-associated zoonoses. PLOS ONE. 2014;9: e91654 10.1371/journal.pone.0091654 24646877 PMC3960114

[11] Bishop JA, Hartley DJ. The size and age structure of rural populations of *Rattus norvegicus* containing individuals resistant to the anticoagulant poison warfarin. J Anim Ecol. 1976;45: 623–646.

[12] Glass GE, Childs JE, Korch GW, LeDuc JW. Comparative ecology and social interactions of Norway rats (*Rattus norvegicus*) populations in Baltimore, Maryland. Occas Pap Museum Nat Hist Univ Kansas Lawrence. 1989;130: 1–33.

[13] Davis DE. The survival of wild brown rates on a Maryland farm. Ecology. 1948;29: 437–448.

[14] Davis DE. A comparison of reproductive potential of two rat populations. Ecology. 1951;32: 469–475.

[15] Gómez Villafañe IE, Cavia R, Vadell MV, Suárez OV., Busch M. Differences in population parameters of *Rattus norvegicus* in urban and rural habitats of central Argentina. Mammalia. 2013;77: 187–193.

[16] Buxton PA. Breeding rates of domestic rats trapped in Lagos, Nigeria, and certain other countries. J Anim Ecol. 1936;5: 53–66.

[17] Indian Plague Commission. The epidemiological observations made by the Commission in Bombay City. J Hyg. 1907;6: 724–798. 10.1017/s0022172400033684 PMC2236259 20474340

[18] Porter FH, Costa F, Rodrigues G, Farias H, Cunha M, Glass GE, et al Morphometric and demographic differences between tropical and temperate Norway rats (*Rattus norvegicus*). J Mammal. 2015;96: 317–323.

[19] World Health Organization. Report of the WHO meeting on rodent ecology, population dynamics and surveillance technology in Mediterranean countries. Geneva; 1992.

[20] Costa F, Hagan JE, Calcagno J, Kane M, Torgerson P, Martinez-Silveira MS, et al Global morbidity and mortality of leptospirosis: A systematic review. PLOS Negl Trop Dis. 2015;9: e3898. 10.1371/journal.pntd.0003898 PMC4574773 26379143

[21] Costa F, Ribeiro GS, Felzemburgh RDM, Santos N, Reis RB, Santos AC, et al Influence of household rat infestation on *Leptospira* transmission in the urban slum environment. PLOS Negl Trop Dis. 2014;8: e3338 10.1371/journal.pntd.0003338 25474580 PMC4256176

[22] Sarkar U, Nascimento SF, Barbosa R, Martins R, Nuevo H, Kalafanos I, et al Population-based case-control investigation of risk factors for leptospirosis during an urban epidemic. Am J Trop Med Hyg. 2002;66: 605–610. 12201599 10.4269/ajtmh.2002.66.605

[23] Felzemburgh RDM, Ribeiro GS, Costa F, Reis RB, Hagan JE, Melendez AXTO, et al Prospective study of leptospirosis transmission in an urban slum community: Role of poor environment in repeated exposures to the *Leptospira* agent. PLOS Negl Trop Dis. 2014;8: e2927 10.1371/journal.pntd.0002927 24875389 PMC4038618

[24] Mills JN, Childs JE, Ksiazek TG, Peters CJ, Velleca WM. Methods for trapping and sampling small mammals for virologic testing. Atlanta, Georgia: U.S. Department of Health & Human Services; 1995.

[25] Sikes RS, Gannon WL, Animal Care and Use Committee of the American Society of Mammalogists. Guidelines of the American Society of Mammalogists for the use of wild mammals in research. J Mammal. 2011;92: 235–253. 10.1093/jmammal/gyw078 PMC5909806 29692469

[26] Leary S, Underwood W, Lilly E, Anthony R, Cartner S, Corey D, et al AVMA guidelines for the euthanasia of animals: 2013 Edition Schaumburg, Illinois: American Veterinary Medical Association; 2013.

[27] Cavia R, Cueto GR, Suárez OV. Techniques to estimate abundance and monitoring rodent pests in urban environments In: Larramendy ML, Soloneski S, editors. Integrated Pest Management and Pest Control—Current and Future Tactics. InTech; 2012 pp. 147–172.

[28] Herbreteau V, Jittapalapong S, Rerkamnuaychoke W, Chaval Y, Cosson J-F, Morand S. Protocols for field and laboratory rodent studies. Chatuchak, Bangkok: Kasetsart University Press; 2011.

[29] Burthe SJ, Lambin X, Telfer S, Douglas A, Beldomenico P, Smith A, et al Individual growth rates in natural field vole, *Microtus agrestis*, populations exhibiting cyclic population dynamics. Oecologia. 2010;162: 653–661. 10.1007/s00442-009-1495-6 19916066

[30] Calhoun JB. The ecology and sociology of the Norway rat. Bethesda: U.S. Department of Health, Education, and Welfare; 1962.

[31] Peig J, Green AJ. New perspectives for estimating body condition from mass/length data: The scaled mass index as an alternative method. Oikos. 2009;118: 1883–1891.

[32] Zar J. Biostatistical Analysis. Englewood Cliffs: Prentice Hall; 1996.

[33] Akaike H. Factor analysis and AIC. Psychometrika. 1987;52: 317–332.

[34] McDonald JH. Handbook of biological statistics. 3rd ed Baltimore, Maryland: Sparky House Publishing; 2014.

[35] Emlen JT, Davis DE. Determination of reproductive rates in rat populations by examination of carcasses. Physiol Zool. 1948;21: 59–65. 18898538 10.1086/physzool.21.1.30151981

[36] Miller N. Reproduction in the brown rat (*Mus norwegicus*). Am Nat. 1911;45: 623–635.

[37] Perry JS. The reproduction of the wild brown rat (*Rattus norvegicus* Erxleben). Proc Zool Soc London. 1945;115: 19–46.

[38] Davis DE, Hall O. The seasonal reproductive condition of female Norway (brown) rats in Baltimore, Maryland. Physiol Zool. 1951;24: 9–20. 14807719 10.1086/physzool.24.1.30152099

[39] Glass GE, Korch GW, Childs JE. Seasonal and habitat differences in growth rates of wild *Rattus norvegicus*. J Mammal. 1988;69: 587–592.

[40] McGuire B, Pizzuto T, Bemis WE, Getz LL. General ecology of a rural population of Norway rats (*Rattus norvegicus*) based on intensive live trapping. Am Midl Nat. 2006;155: 221–236.

[41] Davis DE. The relation between level of population and size and sex of Norway rats. Ecology. 1951;32: 462–464.

[42] Farhang-Azad A. Ecology of *Capillaria hepatica* (Bancroft 1893) (Nematoda). 1. Dynamics of infection among Norway rat populations of the Baltimore Zoo, Baltimore, Maryland. J Parasitol. 1977;63: 117–122. 845721

[43] Davis DE. The weight of wild brown rats at sexual maturity. J Mammal. 1949;30: 125–130. 18935372

[44] Bergallo HG, Magnusson WE. Effects of climate and food availability on four rodent species in southeastern Brazil. J Mammal. 1999;80: 472–486.

[45] Calhoun JB. The social aspects of population dynamics. J Mammal. 1952;33: 139–159.

[46] Blanchard CD, Fukunaga-Stinson C, Takahashi LK, Flannelly KJ, Blanchard RJ. Dominance and aggression in social groups of male and female rats. Bevahioural Process. 1984;9: 31–48. 10.1016/0376-6357(84)90006-8 24923827

[47] Berdoy M, Drickamer LC. Comparative social organization and life history of Rattus and Mus In: Sherman PW, Wolff JO, editors. Rodent societies: An ecological & evolutionary perspective. Chicago, Illinois: The University of Chicago Press; 2007 pp. 380–392.

[48] Costa F, Wunder EA, De Oliveira D, Bisht V, Rodrigues G, Reis MG, et al Patterns in *Leptospira* shedding in Norway rats (*Rattus norvegicus*) from Brazilian slum communities at high risk of disease transmission. PLOS Negl Trop Dis. 2015;9: e3819. 10.1371/journal.pntd.0003819 PMC4457861 26047009

[49] Lantz DE. House rats and mice Farmerʹs Bulletin. U.S Department of Agriculture; 1917.

[50] Costa F, Richardson JL, Dion K, Mariani C, Pertile AC, Burak MK, et al Multiple paternity in the Norway rat, Rattus norvegicus, from urban slums in Salvador, Brazil. J Hered. 2016; 1–6. 26733693 10.1093/jhered/esv098 PMC5893012

[51] Channon D, Channon E, Roberts T, Haines R. Hotspots: are some areas of sewer network prone to re-infestation by rats (*Rattus norvegicus*) year after year? Epidemiol Infect. 2006;134: 41–48. 16409649 10.1017/S0950268805004607 PMC2870354

[52] Davis DE, Hall O. The seasonal reproductive condition of male brown rats in Baltimore, Maryland. Physiol Zool. 1948;21: 272–282. 18871190 10.1086/physzool.21.3.30152004

