# Supplementary figures and images for: A Two-Year Ecological Study of Norway Rats (Rattus norvegicus) in a Brazilian Urban Slum
Source: PLoS One. 2016 Mar 25;11(3):e0152511. doi: 10.1371/journal.pone.0152511 (PMC4807843; doi:10.1371/journal.pone.0152511)

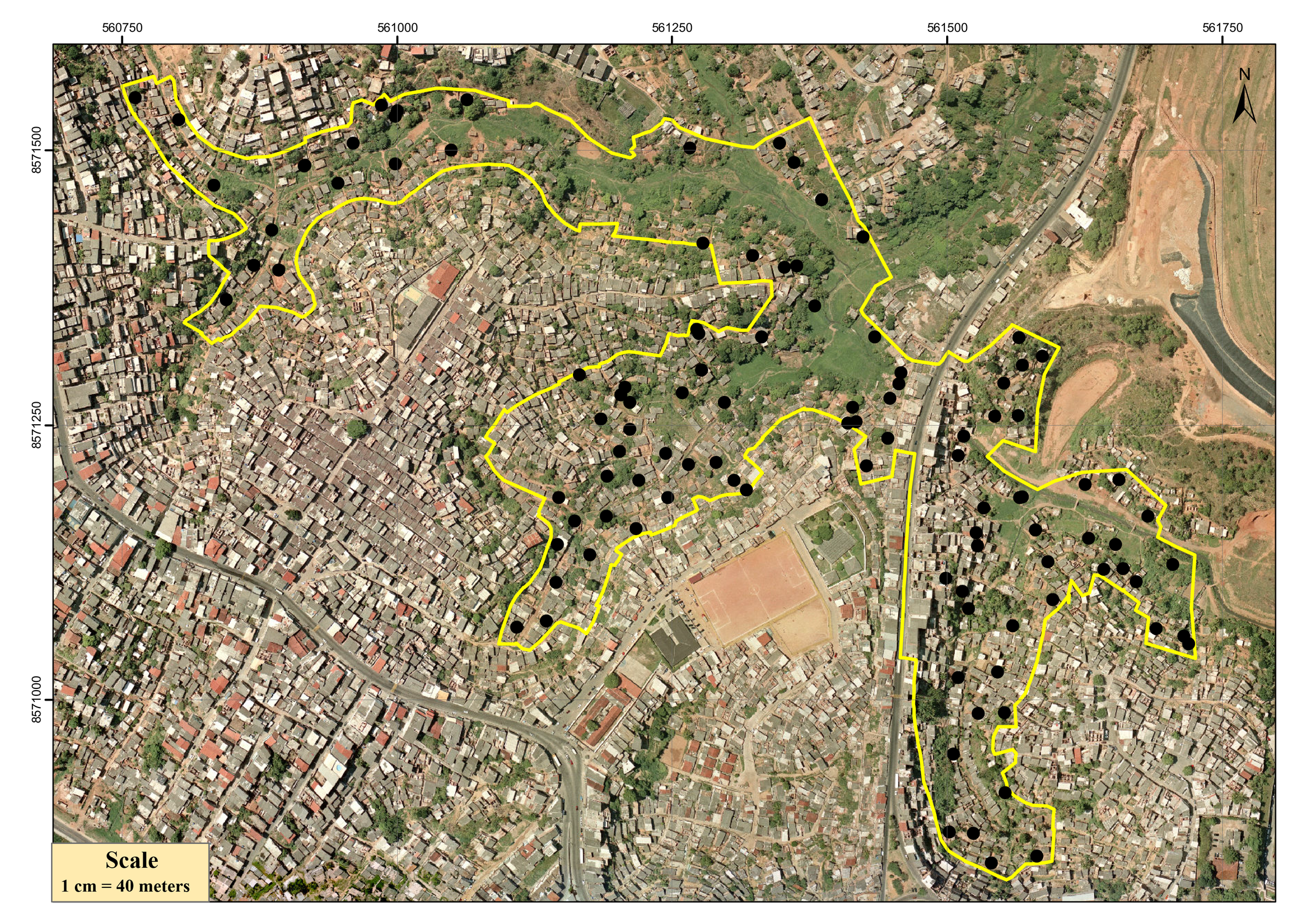

Supplement: S1 Fig — (TIF) [file pone.0152511.s001.tif]
