# Supplementary material for: A Two-Year Ecological Study of Norway Rats (Rattus norvegicus) in a Brazilian Urban Slum
Source: PLoS One. 2016 Mar 25;11(3):e0152511. doi: 10.1371/journal.pone.0152511 (PMC4807843; doi:10.1371/journal.pone.0152511)
Supplement: S1 Table — (DOCX) [file pone.0152511.s002.docx]

| **Characteristic** | **Rainy seasons** | | | |
| --- | --- | --- | --- | --- |
|  | **n** | **TC 1** | **n** | **TC 3** |
| No. of rats |  | 266 |  | 193 |
| Male (percentage) |  | 139 (52.2) |  | 94 (48.7) |
| Female (percentage) |  | 127 (47.8) |  | 99 (51.3) |
| Mean (SE) mass (g): |  |  |  |  |
| Males | 139 | 314 (9.2) | 94 | 295.8 (12.7) |
| Females | 127 | 291.9 (9.9) | 99 | 271.5 (10.8) |
| Mean (SE) age (days) |  |  |  |  |
| Males | 139 | 93 (3) | 94 | 89 (5) |
| Females | 127 | 84 (3) | 99 | 78 (4) |
| Mean (SE) Scaled mass index |  |  |  |  |
| Males | 139 | 227 (6) | 94 | 271 (5)* |
| Females | 126 | 235 (5) | 99 | 264 (5)* |
| No. of sexually active males (percentage) | 138 | 116 (84.1) | 90 | 75 (83.3) |
| No. pregnant rats (percentage)† | 54 | 26 (48.1) | 65 | 34 (52.3) |
| Median (1Q‒3Q) of embryos | 23 | 10 (8‒11.5) | 34 | 10.5 (9.3‒12) |
| No. of lactating (percentage) rats† | 55 | 18 (32.7) | 65 | 23 (35.4) |
| Median trap success (1Q‒3Q) | 9 | 15.4 (12.3‒17.9) | 6 | 11.2 (10.0‒12.9) |

**S1 Table.** **Summary of population characteristics of Norway rats for comparison between trapping campaigns (TC) 1 and 3.**

SE, standard error, 1Q, first quartile; 3Q, third quartile; †, considering only sexually active females; *, P < 0.05.
