# Supplementary material for: A Two-Year Ecological Study of Norway Rats (Rattus norvegicus) in a Brazilian Urban Slum
Source: PLoS One. 2016 Mar 25;11(3):e0152511. doi: 10.1371/journal.pone.0152511 (PMC4807843; doi:10.1371/journal.pone.0152511)
Supplement: S2 Table — (DOCX) [file pone.0152511.s003.docx]

| **Characteristic** | **Dry seasons** | | | |
| --- | --- | --- | --- | --- |
|  | **n** | **TC 2** | **n** | **TC 4** |
| No. of rats |  | 204 |  | 130 |
| Male (percentage) |  | 95 (46.6) |  | 72 (55.4) |
| Female (percentage) |  | 109 (53.4) |  | 58 (44.6) |
| Mean (SE) mass (g) |  |  |  |  |
| Males | 95 | 286.0 (10.7) | 72 | 298.5 (14.3) |
| Females | 109 | 227.9 (9.9) | 58 | 278.7 (14.3)* |
| Mean (SE) age (days) |  |  |  |  |
| Males | 95 | 82 (3) | 72 | 88 (4) |
| Females | 109 | 65 (3) | 58 | 80 (5)* |
| Mean (SE) Scaled mass index |  |  |  |  |
| Males | 95 | 277 (6) | 72 | 284 (6) |
| Females | 109 | 274 (8) | 58 | 272 (7) |
| No. of sexually active males (percentage) | 95 | 78 (82.1) | 71 | 57 (80.3) |
| No. pregnant rats (percentage)† | 52 | 29 (55.8) | 39 | 17 (43.6) |
| Median (1Q‒3Q) of embryos | 29 | 9 (7‒11) | 17 | 12 (10‒13)* |
| No. of lactating (percentage) rats† | 51 | 13 (25.5) | 41 | 22 (53.7)* |
| Median trap success (1Q‒3Q) | 6 | 12.4 (11.2‒13.5) | 6 | 11.5 (10.7‒12.3) |

**S2 Table.** **Summary of population characteristics of Norway rats for comparison between trapping campaigns (TC) 2 and 4.**

SE, standard error; 1Q, first quartile; 3Q, third quartile; †, considering only sexually active females; *, P < 0.05.
