# Supplementary material for: A Two-Year Ecological Study of Norway Rats (Rattus norvegicus) in a Brazilian Urban Slum
Source: PLoS One. 2016 Mar 25;11(3):e0152511. doi: 10.1371/journal.pone.0152511 (PMC4807843; doi:10.1371/journal.pone.0152511)
Supplement: S3 Table — The final models were selected by principle of parsimony. (DOCX) [file pone.0152511.s004.docx]

**S3 Table. AIC values for the four best models for age, scaled mass index (Smi), and wounds.**

| **Model** | **AIC** | **∆AIC** |
| --- | --- | --- |
| *Age in males* |  |  |
| Sexual activity + wounds + Smi | -473.55 | 0 |
| Sexual activity + Wounds | -470.98 | 2.57 |
| Sexual activity + Smi | -424.97 | 48.58 |
| Wounds + Smi | -284.39 | 189.16 |
| *Age in females* |  |  |
| Season + Sexual activity + Wounds + Smi | -348.44 | 0 |
| Season + Sexual activity + Wounds | -346.33 | 2.11 |
| Sexual activity + Wounds | -336.58 | 11.86 |
| Sexual activity + Wounds + Smi | -336.10 | 12.34 |
| *Scaled mass index* |  |  |
| Season + Age | 8334.39 | 0 |
| Season + Age + Sexual activity | 8336.10 | 1.71 |
| Season + Age + Wounds | 8336.4 | 2.01 |
| Season + Age + Sexual activity + Wounds | 8338.1 | 3.71 |
| *Wounds in males* |  |  |
| Age + Sexual activity | 421.33 | 0 |
| Age + Sexual activity + Smi | 423.33 | 2 |
| Age + Smi | 425.79 | 4.46 |
| Sexual activity + Smi | 461.32 | 40.32 |
| *Wounds in females* |  |  |
| Age + Sexual activity | 473.29 | 0 |
| Age + Sexual activity + Smi | 475.10 | 1.81 |
| Age + Smi | 481.27 | 7.98 |
| Sexual activity + Smi | 492.27 | 19.98 |
